# “The co-authors of pregnancy”: leveraging men’s sense of responsibility and other factors for male involvement in antenatal services in Kinshasa, DRC

**DOI:** 10.1186/s12884-017-1587-y

**Published:** 2017-12-06

**Authors:** Michelle M. Gill, John Ditekemena, Aimé Loando, Vicky Ilunga, Marleen Temmerman, Franck Fwamba

**Affiliations:** 1 Elizabeth Glaser Pediatric AIDS Foundation, 1140 Connecticut Ave NW, Suite 200, Washington, DC 20036 USA; 2 Ghent University, Faculty of Medicine and Health Sciences, Sint-Pietersnieuwstraat 25, B-9000 Ghent, Belgium; 3 Elizabeth Glaser Pediatric AIDS Foundation-DRC, 63, Ave. Col. Mondjiba, Commune de Ngaliema, Kinshasa, Democratic Republic of the Congo; 4 University of Kinshasa, Kinshasa School of Public Health, BP 11850 Mont Amba, Lemba, Kinshasa, Democratic Republic of the Congo; 5 Aga Khan University, Faculty of Health Sciences, Limuru Road, East Tower Block, 5th floor, Nairobi, Kenya; 6 National AIDS Control Programme, Kinshasa, DRC, Croisement AV. Des Huileries & Tombalbaye, Concession de l’INRB, Commune de la Gombe, Kinshasa, Democratic Republic of the Congo

**Keywords:** Male involvement, HIV testing, Antenatal care (ANC), Democratic Republic of Congo (DRC)

## Abstract

**Background:**

Despite efforts to improve male involvement (MI), few male partners typically attend antenatal care (ANC). MI in ANC and interventions to prevent mother-to-child HIV transmission have been demonstrated to be beneficial for the HIV-positive mother and her child. This study aimed to explore factors influencing partner attendance and highlight interventions with potential to improve MI within a Congolese context.

**Methods:**

This was an exploratory, qualitative study conducted in two urban and two semi-urban catchment areas of Kinshasa, DRC in June–September 2016. Two women-only and two men-only focus group discussions (FGDs) were held; participants were recruited from ANC clinics and surrounding communities. Key informants purposively selected from health facility leadership and central government were also interviewed. Guide topics included MI barriers and facilitators, experiences with couples’ ANC attendance and perceptions of MI interventions and how to improve them. Data from FGDs and interviews were analyzed to determine three interventions that best addressed the identified MI facilitators and barriers. These interventions were explored further through dialogues held with representatives from community organizations.

**Results:**

This study included 17 female and 18 male FGD participants, 3 key informants and 21 community dialogue participants. Receipt of clinic staff advice was the most commonly-reported factor facilitating male attendance. No time off work was the most commonly-reported barrier. Only men identified responsibility, referring to themselves as “authors of the pregnancy,” and wanting to be tested for HIV as facilitators. The most promising interventions perceived by FGD and interview participants were male partner invitation letters, couple- and male-friendly improvements to ANC, and expert peer-to-peer outreach. Community dialogue participants provided further detail on these approaches, such as invitation letter content and counseling messages targeting men attending ANC.

**Conclusions:**

Common themes regarding male involvement in ANC that emerged from this study included men’s need to understand how the pregnancy is progressing and how best to care for their female partners and unborn children, and ANC settings that were misaligned to the needs of men and couples. Interventions at the individual, facility and community levels were discussed that could result in improvements to male attendance at pregnancy-related services.

## Background

A number of studies have found that male partner involvement in antenatal care (ANC) and interventions to prevent mother-to-child HIV transmission (PMTCT) is beneficial both for the HIV-positive mother and her child [1–3]. Providing suitable medical information to men has several important consequences that foster healthy behavior. First, well-informed men are more likely to participate positively in the decision-making process of the couple [4–6]. Secondly, women with supportive partners are more motivated to undergo HIV testing, to return for the HIV test result and to disclose the HIV result to their partner [5, 7]. Thirdly, well-informed couples are more likely to adopt protective health behaviors (e.g., condom use) and increase mutual support, regardless of the test result [8, 9]. Male partner participation in voluntary counseling and testing (VCT) or attendance at ANC/PMTCT has been associated with increased hospital delivery, uptake of maternal and infant Nevirapine, compliance with breastfeeding guidelines, retention in PMTCT, and HIV-free survival at 6 weeks and following breastfeeding cessation [1, 4, 9–11]. Some benefits were greater when couples were tested and counseled together as opposed to the male partner simply being present at ANC visits [4]. However, one systematic review of male accompaniment in ANC found mixed effects on non-HIV related antenatal and postnatal outcomes [12].

The Elizabeth Glaser Pediatric AIDS Foundation (EGPAF) in the Democratic Republic of the Congo (DRC) has been implementing interventions tailored to improve male involvement (MI) through its PMTCT programs. While the package of services may vary in practice, men attending ANC should be provided with family health education and nutrition counseling alongside their pregnant partners, accompany them during the examination, and be invited to test for HIV. While the HIV prevalence rate in the DRC is lower than elsewhere in the region at 1.2%, 84% of men have never received an HIV test [13]. Interventions to increase male involvement included sensitization campaigns through national media and in the community to encourage male partners to attend ANC services with their pregnant partners and at the facility level, expanding the hours of operation at healthcare facilities, fast-tracking women who attend with their partners, removing signs in facilities that prohibit men, sending invitation letters to male partners of ANC attendees, and training health workers (HW) on male partner sensitivity in service delivery. These interventions have not been consistently implemented in the DRC and more understanding of how these strategies influence male behavior is needed. Despite the multiple approaches to improving male involvement, low male participation remains a challenge. Approximately 7% of male partners attended maternal and child health (MCH) activities in EGPAF-supported facilities from October 2015 to June 2016 [14].

Rates of MI are also low elsewhere in sub-Saharan Africa; the proportion of men participating in ANC/PMTCT ranges from 5% to 20% [11, 15–17]. This may be explained in part by the multitude of well-documented barriers to MI. Male partner involvement is limited by socio-economic (e.g., men’s work and other commitments) and socio-demographic (e.g., age) factors and attitudes, such as fear of knowing one’s HIV status, men’s lack of knowledge or interest, and women’s unwillingness due to fear of a negative partner reaction resulting from his involvement in ANC/PMTCT. Interpersonal factors (e.g., relationship conflict, lack of communication within the couple, unplanned or extramarital pregnancies), health system factors (e.g., treatment of pregnant women, men prohibited from ANC, fees, unwelcoming and inadequate space, lack of privacy, HW attitudes, limited staff), and cultural issues and norms (e.g., perception that involvement in ANC undermines men’s position, pregnancy considered women’s domain) have also been noted [6, 16, 18–20].

While literature is replete with factors influencing partners’ attendance, a greater understanding is needed of the barriers vis-à-vis specific MI intervention strategies and facilitators that can be leveraged to help improve male engagement, particularly in settings such as the DRC, where some efforts have been made without significant shifts in behavior. The aim of this study was to explore the facilitating and inhibiting factors influencing partner attendance at antenatal services identified by pregnant women, men of reproductive age, public health leaders and community members in order to help explain why aspects of selected MI interventions may or may not be effective. Three interventions were highlighted for further evaluation of their potential to improve MI within a Congolese context.. While multiple definitions of male involvement in MCH have been used in the literature, this study focused on specific MI interventions related to male attendance at ANC.

## Methods

This was an exploratory qualitative study. Data were collected through focus group discussion (FGD), key informant interview (KII) and community dialogue (CD). Four health facility catchment areas in EGPAF-supported health zones in Kinshasa with high volume in ANC (for ease of recruitment) were purposively selected for this study. Facilities with inaccessible roads at some times during the year were excluded.

One women-only FGD each was conducted in an urban and semi-urban catchment area. One men-only FGD each was also conducted in an urban and semi-urban catchment area, for a total of four FGDs in four different areas. Women were eligible if they were currently pregnant and attending ANC services at one of the study facilities on the day trained study personnel were present; any primigravida who were attending a first ANC visit were excluded as their exposure to MI interventions in ANC would be negligible. Eligible women were referred by clinic staff to the study team consecutively for recruitment until approximately eight women agreed to participate. Men 18 to 59 years of age who were living in one of the study facility catchment areas and who had at least one child born in the last 3 years and/or who had a currently pregnant female partner were recruited from ANC clinics and the surrounding communities in order to reflect the experiences of those who have and have not attended ANC. They were recruited from venues in the communities (described below) frequently attended by men by a member of the study team following introductions by the person in charge of the venue. Approximately two men were invited to participate at each venue and were provided with a date and location for the FGD; men were enrolled when they presented for the discussion.

Three key informants were purposively selected for interviews based on their position to inform policy or programmatic change with regards to MI or their demonstrated experience and knowledge of MI interventions. Two central Government representatives of HIV and reproductive health programs and one health care provider from one of the study facilities in the four catchment areas were recruited and participated in interviews.

Two CDs took place in two of the study catchment areas: one urban, one semi-urban. A sampling frame was developed which included community organizations within the area. Organizations were purposively selected in order to have various professional, community development, athletic, and religious organizations represented. For each dialogue, invitation letters were sent to ten organizations requesting participation from one representative in order to solicit their feedback on approaches to increase MI in pregnancy-related services.

FGDs and KIIs took place in June 2016 and the CDs were conducted in September 2016 by trained study staff. Focus groups and dialogues were conducted in Lingala and the interviews were conducted in French; all sessions were audio-recorded. All participants provided informed consent prior to any data collection and completed a brief demographic form tailored to the participant group. For instance, FGD participants were asked about their number of children and HIV testing history. Key informants were asked for their number of years of professional experience and dialogue participants were asked about their number of years living in the community. Recordings were transcribed verbatim and translated into English for analysis.

The FGD and KII guide topics included facilitators and barriers to men’s involvement in ANC/PMTCT, experiences with couples’ ANC attendance, and advantages and disadvantages of existing MI interventions at Kinshasa facilities and interventions proposed for the DRC context. Interventions in which participants may have been exposed were included in order to capture perspectives on the extent to which they encourage men to attend services and how they could be improved. Proposed interventions were determined by investigators based on the literature. Any adaptations or other recommendations for interventions were also sought.

FGD and KII transcripts were first read carefully by four investigators. Unclear or ambiguous segments of text were identified and discussed to reach consensus on meaning or to clarify translations. FGD and KII data were categorized according to an a priori codebook, with codes derived from the interview guides (e.g., facilitating and inhibiting factors, interventions). Codes were added that emerged from the data (e.g., facilitating and inhibiting factor sub-codes). Data were coded using the qualitative software program, MAXqda (V10). Coding reports were then reduced to matrices and textual summaries organized by themes (the topics listed above) and were reviewed by investigators. Quotes that illustrated the findings were identified. Thematic content analysis was used to identify similarities and differences among women, men and key informants and overall patterns in the data [21].

As part of this review of the FGD and KII data, the same investigators also collectively determined the three most favorably-perceived interventions that best addressed the identified facilitators and barriers to MI. These interventions were explored in-depth through facilitated discussion in the CDs according to a loosely structured tool with the following questions: 1) likes/dislikes of the intervention; 2) its effectiveness at promoting MI; and 3) suggestions to make the intervention more acceptable, feasible or effective. The two transcripts were read carefully by investigators. Responses from both CDs were summarized with illustrative quotes and organized under each question. Then, inductive codes were created and manually applied for a more in-depth analysis of specific aspects of these interventions. For instance, codes for suggestions to improve the ANC setting/services included friendlier clinic staff, male-specific counseling and physical changes to facilities. These data were triangulated with the FGD/KII responses on the interventions as well as any data that emerged from the dialogues on facilitating and inhibiting factors (though this was not the focus of the CDs).

## Results

### Demographics

There were 8–10 participants in each of the four FGD for a total of 35 participants (17 women and 18 men). Men were recruited from ANC clinics (*n* = 3), religious congregations (*n* = 3), football clubs (*n* = 5), community-based organizations (*n* = 4) and bars (*n* = 3). Women were younger than men with a mean age of 29.4 years and 41.1 years, respectively. The majority were married (*n* = 33, 94.3%). The number of pregnancies among women ranged from two to six, while men had between one and eight children. One woman versus ten men had never been tested for HIV. Of the eight men who had been tested, two were tested during ANC and seven were tested ≥12 months prior to the interview. All men and women tested were HIV-negative.

One male and two female key informants were interviewed, with backgrounds in public health and medicine. The number of years worked in their current organization or facility ranged from 6 to 28 years. The health facility manager was based in a semi-urban catchment area.

The mean age of CD participants (*n* = 21) was 40.4 years and had spent an average of 18.4 years in the community where the dialogue took place. Five participants were female (23.8%). Representatives were from the following organizations: professional (*n* = 2), community development (*n* = 12), athletic (*n* = 2), religious (*n* = 2), and other (*n* = 3). There was one member from each organization represented in the community dialogues, except for one community development organization which had two representatives in one dialogue.

### Factors influencing male involvement

#### MI facilitators

The most frequently cited motivating factors in FGDs and KIIs were 1) a sense of responsibility for the pregnancy and unborn child; 2) desire to understand the progression of partner’s pregnancy and receive counseling from clinic staff; 3) mother or child health problems requiring urgent attention; and 4) to be tested for HIV. While CD participants were not specifically asked about influential factors, some of their perspectives also emerged from the dialogues and are included here.

A sense of responsibility was only cited by men and in KIIs and was a cross-cutting theme that was raised as men discussed other motivating factors, such as the love of their wife and child.

*What drives a man to accompany his wife to the ANC, is primarily a matter of responsibility… because in reality it is the man who is the author of the pregnancy. (Male FGD participant)*

*What encourages me is this love for my wife and the love I have towards this future child I would like to see born without problems. So, that's why I want to follow my wife and accompany her to the maternity for ANC. We want to know everything, to see the doctor, to ask [about] the development of the child, how my wife is progressing, etc. It is this love that I have for her. (Male FGD participant)*

A few men spoke about the need to ensure their female partners took proper care during pregnancy. These responses ranged from women requiring support or guidance from partners on health-related matters to believing that women may be negligent about pregnancy-related care.

*…My daughter…was born with malaria because my wife had been prescribed the drugs but did not adhere to treatment. I was saying, ‘how does it affect me?’ I was not aware of what would come because I was not informed of bad consequences. If I were involved from the beginning I would have assisted my wife to adhere to the treatment. (Male CD participant)*

Finally, female and male respondents mentioned that men may prefer to pay the facility directly for ANC services instead of female partners attending services on their own and being provided with the necessary payment, to ensure money was being spent as intended on ANC.

Receipt of advice or counseling by clinic staff motivated men to be involved in ANC services. Some men wanted to receive information directly from the source. Men indicated they were more likely to heed the advice of a doctor rather than the woman relaying the doctor’s message. They believed clinicians may also prefer to talk to men, particularly if there is a problem. The trust men placed in medical professionals is exemplified by this quote:

*I really see that it is important to talk to doctors like you. If a doctor opens his door,…you learn things. We cannot go elsewhere. We will still return to him because it is he who is the god who can heal people’s lives. (Male FGD participant)*

Men also seemed to be motivated to attend when there was a serious health problem afflicting the mother or child. Some women said that their male partners would only attend in an emergency situation. However, the need to know in general how the pregnancy is progressing was just as frequently mentioned, suggesting men do want to be involved in more of the routine details and to be reassured of the mother and child’s health.

While key informants offered few motivating factors for ANC attendance, they also identified men’s desire for information and sensitization, closely linked with the desire to pay close attention to the pregnancy.

*This is what makes the husband go [to clinic], he always wants to know how the services are organized, what steps are taken to assist pregnant women from the first visit until delivery. When he enters, we first begin with that. We explain to him that there is a particular problem or that there is no problem and that the [pregnancy] is developing well. (KII)*

The desire to be tested for HIV was mentioned by men only as a reason to attend ANC, though several women described that their husbands accepted the test easily.

*If the doctor sent me an invitation, I cannot refuse to answer. I'll be going to know my HIV status. (Male FGD participant)*

Most of those who raised this issue did so because they reasoned that it was important to know the status of both parents, in order to know how to manage the pregnancy and protect the child.

#### MI barriers

The most common factors inhibiting male involvement from the FGDs and KIIs were 1) lack of time, typically due to work commitments; 2) a clinic environment unwelcoming to men and couples; 3) pregnancy considered to be the woman’s domain; and 4) men’s fear of HIV testing.

Respondents from FGDs and KIIs, but women more frequently than men, explained male partners could not attend because their work schedules would not permit: they work 7 days per week, were not off work during clinic hours, or were too tired to attend services after working all day, particularly since attending ANC typically involves significant waiting time. A couple of other men indicated that this was used as an excuse for the actual reasons, such as a fear of HIV testing.

An unwelcoming antenatal clinic environment was also cited frequently by all respondents. In ANC, some respondents described a poor reception by HW, a tendency to neglect couples and the restrictive set-up of clinics. Waiting areas were already crowded with limited seating and men were often asked to sit elsewhere or wait outside.

*I went along with my wife and the doctor spoke with her and left me outside…[when] it finished, we went home…I expected that as I went along with my wife, the doctor would explain to me, for instance, the development of the pregnancy …We arrived at ANC [and] the man was not considered. (Male CD participant)*

Some felt that since only pregnant women may enter ANC, it is useless for men to attend only to wait outside. Other men and women argued that men were not prohibited in hospitals, likely just the delivery room; if anything, they received privileged treatment in these settings. The key informant managing a health facility believed it was a supportive environment for men. A couple women did not want men involved. As one explained,

*For me, ANC is only for women; leave male partners out of it. Leave them the burden of earning money to help the family, and my pregnancy until the day I give birth is my concern. (Female FGD participant)*

Pregnancy and related care perceived as the woman’s domain was mentioned more frequently among men. This was reflective of Congolese culture to not accompany women to ANC and norms which dictate when and how men and women typically interact. Several male respondents indicated that they were uncomfortable in places with many women, such as ANC, where they discussed topics that men found annoying or unrelatable.

A fear of or reluctance to HIV testing was another challenge to men’s involvement in ANC. FGD and KII respondents explained that husbands do not want an HIV test and will just assume their female partner’s negative HIV result applies to them as well. Another key informant felt that men who were tested during a past pregnancy would not want to attend ANC to be tested again.

### Proposed and existing interventions

FGD and KII participants’ feedback on MI interventions are organized below according to individual/couple, facility and community levels. Table 1 includes the three interventions perceived most favorably by respondents: male partner invitation letters, male- and couple-friendly improvements to the ANC setting and services, and expert peer-to-peer outreach. The interventions are linked to the common facilitators and barriers identified by FGD and KII participants. CD responses from these three interventions only are also included below.

**Table 1:** Proposed interventions linked to factors influencing male involvement

Proposed Intervention	Addressing facilitators	Addressing barriers
**Male partner invitation letters**		
Content to include men’s responsibility as the father and encourage attendance based on love for his family to help ensure a healthy pregnancy	• Sense of male responsibility • Desire to understand progression of partner’s pregnancy and receive counseling from clinic staff • Mother or child health problems requiring urgent attention	• Pregnancy considered to be the woman’s domain • Men’s fear of HIV testing
Content to include men’s need to understand how the pregnancy is progressing and what to expect during delivery		
Tone should convey importance without being alarmist (though criticality should be communicated if appropriate)		
Letter should be written by the doctor and highlight her/his credibility in order to convey authority		
HIV should not be specifically mentioned		
**Male- and** **couple-friendly** **improvements to ANC**		
Male-specific services and counseling or greater involvement of men in ANC	• Desire to understand progression of partner’s pregnancy and receive counseling from clinic staff • To be tested for HIV	• Lack of time/work commitment • Clinic environment unwelcoming to men • Pregnancy considered to be the woman’s domain
Reduce time spent at clinic		
Separate spaces for couples with available seating		
More attentive health workers, sensitive to the needs of couples		
**“Expert”** **peer-to-peer** **outreach**		
Help men to understand the importance of attending ANC and what to expect during delivery	• Sense of male responsibility • Desire to understand progression of partner’s pregnancy and receive counseling from clinic staff	• Lack of time/work commitment • Clinic environment unwelcoming to men • Pregnancy considered to be the woman’s domain
Peers will serve as a conduit to the health facility and refer men to clinicians for counseling and services		
Offer men an opportunity to discuss pregnancy and other health-related issues outside the clinic and without women present		

#### Individual/couple level interventions

##### Male partner invitation letters

Several FGD and CD respondents described their experience with this approach. They felt it could encourage male attendance and there were few negative aspects highlighted. A male FGD participant and key informant noted that men may ignore these invitations for reasons such as lack of time. A couple CD participants warned that women may not give the letter to her partner and recommended that men’s attendance should be mandated, not requested. Most agreed that HIV testing should not be mentioned in the letter. In particular, letters were thought to be an opportunity to address any problems with the pregnancy and highlight the importance of protecting the child’s health, to counteract the perception that men were invited to ANC solely for HIV screening. The letter would also capitalize on the gravitas of the doctor as an expert, as male and female respondents agreed that a doctor’s invitation could carry more weight than a man’s pregnant partner. A male FGD respondent also suggested that invitation letters should be preceded by outreach campaigns, similar to what is done for immunizations, to help foster a sense of men’s responsibility.

##### Incentives for women accompanied by male partners

While key informants were more open to the idea of monetary incentives provided to women who bring their male partners to ANC, many participants viewed this strategy negatively. FGD participants felt that men who attend clinic do so out of a sense of responsibility, because they are motivated by concerns over their wives and children’s health, not by what they described as a pay-off.

*“What I am arguing is the sense of responsibility. But the question we must ask is, ‘why we must we give money for the man to just show up?’…It may be that we have invited the man several times, but he does not attend, arguing a lack of money to pay for transport. Finally, you have to [give] money to get him. But I do not support giving you money for your own enjoyment. We need men who really have a sense of responsibility.” (Male FGD participant)*

Moreover, incentives may not overcome the identified barriers, such as the perception that pregnancy and ANC are the woman’s domain and time off work. Sustainability of the approach was a concern in the male FGDs and among key informants, including one who referred to incentives as “bait” and explained that the focus should be on changing people’s conscience, not buying services. Another key informant pointed out that the funding structures of implementing partners would make it difficult to guarantee support for such initiatives indefinitely. FGD participants suggested interventions addressing poverty, such as employment or income generation activities, could be more effective.

##### Reimbursement for travel costs to clinic

There was also some resistance to this strategy due to concerns over sustainability and funding, similar to the reasoning against incentives. It was described as payment for something that men should be compelled to do anyway. However, respondents seemed to be comparatively more open to this approach. In two FGD (one male, one female), it was suggested that transport reimbursement does not need to be promised in advance or consistently provided, it can depend on circumstances. A question also arose on how it could be applied equitably to clients, for those who live in close proximity to the clinic versus further away from clinic. A key informant reported that such a strategy had been implemented in the past with some success. The suggested amount to be offered as reimbursement varied: one said he would be happy with any amount, another proposed an amount equivalent to the cost of delivery and a third respondent said the amount could increase for each subsequent ANC visit attended, first to the man, then to the couple.

#### Facility level interventions

##### Expanded clinic hours

FGD participants were not aware of this intervention, but since work and time were cited frequently as barriers to ANC attendance, they felt such an intervention could help to address this challenge. No one strongly opposed the intervention, though there were some concerns about the possible implications for clinic flow, that even with extended hours, some male partners still would not be able to attend and the costs to cover overtime for HW. There was disagreement among participants over whether a Saturday clinic or late hours during the week would result in greater male attendance. One man suggested there should always be ANC nurses on call.

##### Fast-tracking couples at ANC

This intervention was also felt to be encouraging for men who could not attend ANC due to work or other commitments. More men seemed to be in favor of the strategy, though both men and women acknowledged that it is unfair to those whose husbands truly cannot miss work to attend clinic and for women who do not have partners. The key informants recognized this issue as well, but noted success with the approach regardless. They reasoned that women attending without male partners may feel aggrieved, but it can be used as further encouragement to get them to attend as a couple for the next ANC visit. One had suggested organizing services into two rooms: one for women accompanied by their male partners and one for women attending without a male partner.

##### Male- and couple-friendly improvements to ANC

FGD and KII respondents agreed that antenatal clinic changes could both motivate men to attend as well as be a necessary accompaniment to other MI initiatives to accommodate an influx of male attendees. CD participants recommended a reorganization of clinic flow to reduce the time men would have to spend at ANC, a separate area for couples at clinics with clean, comfortable, and available seating and more attentive HW who are sensitive to the needs of couples and would provide a warm welcome at reception. Their other suggestion was to have some counseling messages tailored to men. This included non-HIV-related health information, such as malaria prevention and family planning, how to help their female partners manage pregnancy and health, any potential hazards to the pregnancy and finally, the benefits of MI, to make it more likely men will return to ANC.

#### Community level interventions

##### Expert peer-to-peer outreach

When asked what strategy could have the greatest potential for improving MI in the FGDs and KIIs, some form of community outreach was the most common response. In particular, peer education and outreach was a positively regarded strategy, in most FGDs, the KIIs and CDs, though one female FGD was skeptical this approach could work. A male FGD participant justified his support of peer interventions:

*Indeed, when men find themselves outvoted in a room full of women, some speak without holding on, they will be embarrassed. It is therefore preferable that they are together, talking about topics that interest them.*

In this intervention, men who had previously been involved in ANC services, would be trained on MCH and MI issues to become “experts” and would then relay information to other men. In addition to peer sensitization, men could also trace those couples who were lost to follow-up from pregnancy-related services. Participants defined these experts as opinion leaders, men who were married, responsible, serious, and competent communicators. Suggested messaging could encourage men to take responsibility starting in early pregnancy, follow the instructions of the doctor and accept HIV testing. Proposed settings in which outreach could take place included work places, schools, sporting events, association meetings, and under trees in neighborhoods where men play games. Respondents were conflicted about whether or not bars and entertainment clubs would be a conducive place for these exchanges. It was argued that while these venues promote drinking and sometimes illegal activity, they could be an effective platform to reach youth. Holding awareness-raising days or establishing outreach centers throughout Kinshasa so information can be provided at any time was also suggested.

##### Mass media campaigns

There were limited insights offered on mass media campaigns, which came largely from the KIIs. These respondents saw positive aspects with this approach, but generally felt that there were more effective ways of disseminating information to encourage MI. The disadvantages were that it can be a passive approach; it is considered expensive and thus, not sustainable; and no one medium could reach everyone. For instance, men and key informants both pointed out that billboards and posters require literacy, while radio or TV require access and electricity. Here is the downside that one key informant described:

*Because I think that mass media activities do not have a great influence on the people. But interpersonal activities really have influence…Yes. It means somebody is near you and can ask questions of clarification if necessary and receive an answer. But when it is via media, when am I going to reach you to ask questions? It’s a bit complicated with mass media, but interpersonal activities are more effective.*

## Discussion

While men in this study acknowledged their belief that ANC is the woman’s domain, many indicated a sense of responsibility regarding their partners’ pregnancies. Common themes regarding male involvement in ANC that emerged from this study included men’s need to understand how the pregnancy is progressing and how best to care for their female partners and unborn children, as the ‘co-authors’ of pregnancy, and ANC settings that were misaligned to the needs of men and couples. Male partner invitation letters, improvements to the ANC setting and services to make the clinic a more welcoming environment for men and couples and expert peer-to-peer outreach, were discussed as potential interventions at the individual, facility and community levels, respectively, to improve male attendance at pregnancy-related services.

That men were responsible for their unborn child was provided as justification for being involved in ANC. However, elsewhere male respondents maintained pregnancy and child birth were women’s responsibilities [22]. The belief that pregnancies are the primary purview of women is perpetuated and reinforced when the invitation to attend ANC or get tested for HIV comes from their female partners and by the female-oriented setting in which these services are provided [23].

While male partner invitation letters have been used in some Kinshasa facilities already, it was still viewed as a favorable MI strategy in this study, with some notable suggestions for improvement. These included 1) highlighting the expert opinion of the clinician, 2) stressing the responsibility of men to be involved in the health of their partners and children, and 3) conveying the urgency of their attendance without being alarmist. MI rates rose to 16% in Uganda and to 54% in Tanzania following the use of letters [6, 8]. Letters also increased the likelihood that the partner would attend by 50% compared to women’s verbal invitation alone [24]. The invitation letters in Tanzania which resulted in a dramatic change from pre-intervention rates of MI, was signed by the regional medical officer and did not reference HIV. While HIV testing was noted here as both a barrier and motivating factor to MI, most respondents were in agreement that HIV should not be mentioned in the letter. However, Mohlala et al. found that an invitation letter inviting men to ANC plus VCT was more effective than an invitation letter to ANC plus a pregnancy information session [25]. Moreover, the main reasons cited by male partners accompanying women to ANC were wanting an HIV test and health information [26]. Other studies have supported the finding that an invitation from clinic staff would carry more weight than from the female partner [23, 27, 28]. When the onus is on women themselves, it requires being empowered with the right information and the ability to encourage men to attend. Finally, an invitation espousing fatherhood-related themes was also felt to increase the likelihood that men would accompany their pregnant partners to PMTCT [29].

Respondents in this study also offered recommendations for making ANC clinics more conducive to the needs of men and couples, with a friendlier reception by HW, more comfortable waiting areas, and services which offer men reasons to attend other than to get tested for HIV and to accompany their female partner. Previous facility-level strategies in Kinshasa have largely lacked a package of services to offer men in ANC or standards for addressing couple’s needs. Factors such as education sessions delivered in departments other than ANC, private rooms for couples counseling and other PMTCT interventions, preferential treatment for couples attending together, and positive HW attitudes have been reported to facilitate MI [19]. Some gains in MI were found in Malawi following a clinic expansion that included a larger waiting area for antenatal attendees, bigger spaces for HIV couples counseling and testing, and the addition of men’s toilets [30]. Montgomery et al. proposed a greater focus on men’s health needs, more than just encouragement to participate in the health of their female partners [31]. Our study offers some ideas for male-specific counseling but more research is needed on what services could be best combined with women’s antenatal care. For instance, most men had attended clinic for STI testing when notified that their partner had an infection, but then did not return to ANC thereafter [28].

There is evidence that varying forms of community sensitization and/or mobilization are effective at encouraging male attendance at ANC and delivery. This includes peer education through drama, male peer individual or group initiatives, safe motherhood campaigns, and involvement of influential leaders [19, 28, 30]. Linking “expert” male peers in order to encourage other men to become involved was discussed in this study, which has the potential to address the barrier that ANC is perceived to be the women’s domain. It could also provide a platform to disseminate information to men on how to monitor the pregnancy and prepare for their child’s birth and how to support their partner on a schedule that could work around their other commitments. CD participants supported this approach. Mentor Mothers, a strategy already established in Kinshasa and other DRC provinces, in which expert mothers assist newly identified HIV-positive pregnant women to navigate the PMTCT program and who follow up with women who miss appointments, offers a model for a male version which could be implemented and evaluated [32]. It should be noted that successful approaches were rarely singularly focused and increasing male attendance in ANC and HIV testing found in other studies often involved interventions coupled with a community-based intervention [6, 7, 30, 33].

This exploratory study has limitations. We had a small number of FGD and KII with differing characteristics (male, female, key informants). Given the sample size, heterogeneous groups and the exploratory design of this study, some of the impressions and experiences related here may have only been expressed by a small number of participants and we were not able to draw conclusions regarding the effectiveness of these approaches. However, we were able to add depth to the analysis by triangulating data from the additional men and women participating in the CDs. They lived in the same communities and had their own interactions with pregnancy-related services as evidenced from their responses, but spoke about the feasibility and potential usefulness of strategies in promoting MI based on their position as community leaders and representatives. Secondly, we did not specifically target HIV-positive women or men. None of our sample was HIV-positive and people living with HIV may have different perspectives and experiences. However, the prevalence of HIV in the DRC is lower compared to other countries in the region and male attendance at ANC is important for many reasons, including to help ensure HIV-negative couples remain un-infected. Women were also younger than men, which was likely due to the narrower reproductive age range of women, though such an age difference is not uncommon among couples in Kinshasa and elsewhere in the DRC. Finally, FGD participants had varying levels of direct exposure to interventions discussed, though by not controlling this factor, we were able to elicit their feedback on numerous interventions, which is one of this study’s strengths. While many published MI studies have focused on one or a few interventions, this study offers additional insight on several interventions as to why they may or may not be effective in this context.

## Conclusions

Our study moved beyond an identification of MI barriers and facilitators by eliciting feedback from program recipients and planners on existing and proposed MI interventions and refined these strategies in community dialogues. This study highlighted interventions and specific components at the individual/couple level (male partner invitation letters), facility level (changes to ANC to make a more welcoming environment for men) and community level (expert peer-to-peer outreach) that should be evaluated for their feasibility and effectiveness at improving MI within Congolese and similar contexts. Despite the barriers to involving men in antenatal care, this study also identified factors, such as men’s sense of responsibility and desire to be involved, that could be leveraged in order to engage more men in pregnancy-related services in order to improve the health of women, children and their partners.

## Abbreviations

ANC: Antenatal care
CD: Community dialogue
DRC: Democratic Republic of the Congo
EGPAF: Elizabeth Glaser Pediatric AIDS Foundation
FGD: Focus group discussion
HW: Health workers
KII: Key informant interview
MI: Male involvement
PMTCT: Prevention of mother-to-child transmission
SD: Standard deviation
VCT: Voluntary counseling and testing
